# A Drop of Water

**Published:** 1869-04

**Authors:** J. D. Steele


### A Drop of Water.

BY PROF. J. D. STEELE.

Science teaches us to watch for the beautiful in the homely. The ocean is a grand object; mountains are sublime, but to be thrilled with elevated thoughts one need not go to the sea shore or visit Switzerland. Balance on your finger a drop of water, and gazing into its crystal depths with the eye of philosophy, you can see a world of beauty and grandeur. The drop seems quiet and lifeless, yet in its transparent cells are locked an amount of electric force equal to that of a thunder bolt. It is not "dumb mineral matter," as we often call it. The rather is it instinct with power. All the forces of Nature cluster about it. Gravity draws it towards the earth. Chemical affinity binds together the atoms of oxygen and hydrogen of which it is composed. Cohesion rounds it into a globe. Heat keeps it in a liquid form, and Adhesion causes it to cling to the finger.

That drop of water is a part of a circulation vast as the world. As has been beautifully said,—Water is the blood of the earth. The ocean is the heart; clouds and vapors are the arteries; the rain streams, the capillaries nourishing every corner, and the brooks and rivers the veins which return the life-current to its source. Water washes the mineral matter of the mountains into the valleys to fertilize them. It dissolves the elements of the soil and climbs up into the leaf, to be there worked up into the structure of the plant. It cleanses the air, keeping it pure and invigorating. It washes the face of Nature, and makes good old mother earth clean and presentable. It ebbs to and fro through our bodies, bearing the materials of growth and life. It is the grand agent in geologic changes. Here water scoops out a valley, there it builds up an island from the foundation, and yonder it buries one in the depths of the sea. The broad valley where stands Elmira was dug out by the Chemung in the far away past when it was a mighty river sweeping southward with a majestic flow like that of the St. Lawrence. To perform this work water requires to be in constant circulation. It is never at rest. A drop may at one moment float in the depth of mid-ocean, at another on the ladder of the sun-beam mount to the clouds. It flies on the wind, falls in the rain, thunders in the cataract. Now it is piled on the mountains as snow; now it is gathered in Alpine glaciers. Now the sun unlocks its icy fetters, and away it hurries to its old home in the sea. Below, the rivers flow constantly toward the ocean, but above, high in air, are invisible rivers of vapor that move back to the mountains and feed the sources with a constant supply.

The various forms and adaptations of water are wonderful indeed. It is fitted into the economy of Nature like a cog into a wheel. It does not possess a property which does not in some way minister to our necesities and comforts.—

Let us notiee a few only, of these striking proofs of Divine forethought and care.

In the intense sun-beam of the tropics, the water of the ocean becomes highly heated. A portion is thus evaporated and ascends to the upper regions of the atmosphere, while another portion is simply expanded and rises to the surface. The former part is floated by the wind to distant regions. Becoming gradually chilled, the vapor is condensed and the water showered down in warm fertilizing rains. The heat which the vapor absorbed in the sunny South is thus set free to temper the severity of our snowy climate. This constitutes a natural steam apparatus on the grandest scale, since it has a boiler at the equator and steam pipes traversing the entire globe The latter portion establishes warm currents of water which flow also Northward. Thus the Gulf Stream sweeps the warm waters of the Caribbean Sea along our Eastern Coast across the Atlantic from Cape Fear to the western shore of Ireland. Starting with a temperature of 83 degrees, it warms our eastern harbors, redeems England from the cold of Labrador, which lies in the same latitude, saves the Tiber from the wintry ice of the Hudson and cheats sunnny Italy out of thesleigh-rides of New England. Should it by any chance break through the Isthmus of Darien, Great Britain would be condemned to eternal glaciers. Water is thus a grand magazine and distributor of heat. Its constitution peculiarly fits it for this purpose. To elevate the temperature of a lb. of water one degree requires thirty times as much heat as for a lb. of mercury. You know how long it takes to boil a kettle of water, even over a brisk fire; the stove will often become red hot long before the kettle begins to sing. In turning to vapor it absorbs over 900 degrees of heat. This great amount becomes latent or concealed in the hot water or steam and is given up again when it is cooled. This property enables water to temper the transitions of the seasons and to equalize the climate. In the summer our pond and lakes absorb vast quantities of heat. As the winter approaches, the water cools, and before it freezes gives up this great store of heat which it has so greedily hoarded. In the spring the melting ice and snow drink in the sun-beam which else might prematurely coax forth the tender buds to be blighted by untimely frosts. Since, too, so much heat is required to melt the ice and snow, they dissolve very slowly and thus prevent in a measure those devastating floods which would inevitably ensue on the first warm day, if ice should melt as easily as many other solid bodies.

Water contains air which is necessary for the support of the fish which inhabit its crystal domains. This air, however, subserves another most important use. Water, when deprived of air does not boil like common water at 212 degrees F., but rises in temperature often as high as 290 degrees, and then bursts at once into steam with explosive violence. Thus, were it not for the air always present in water, every stove boiler would require a thermometer. To run a tea-kettle as careful watching would be necessary as now to attend a steam engine, and our kitchens would be made to witness frequent and most disastrous explosions. As it is now, as soon as the temperature rises above 212 degrees, the extra heat passes off quietly and safely.

Water in all its varied forms is a "thing of beauty." As frost, it decks the barren trunks of trees, the homely spires of grass and the commonest window pane with crystals of exquisite delicacy and grace. As dew, it sparkles in the morning sun like the rarest gems. As clouds, it floats high in air, pure as "the white robes of saints," or gathering about the horizon, paints the sky with colors which no human art can equal. In cataracts, mountain torrents, glaciers, bubbling fountains, placid lakes and murmuring brooks, it gives to the landscape animation and life.

Water symbolizes thought. The brook that chatters so noisily, the drops that patter so merrily, the broad flowing river—the emblem of life, the ocean—the symbol of eternity, the falling tear—the token of grief, the baptismal font—the seal of purity, and the bow—the pledge of mercy, all illustrate the beautiful truths that cluster around this precious gift of God.

---

What disorder excites the greatest compassion? The small-pox, for the patient is generally pitied (pited.)

